# Association of objectively measured arm inclination with shoulder pain: A 6-month follow-up prospective study of construction and health care workers

**DOI:** 10.1371/journal.pone.0188372

**Published:** 2017-11-27

**Authors:** Markus Koch, Lars-Kristian Lunde, Kaj Bo Veiersted, Stein Knardahl

**Affiliations:** National Institute of Occupational Health, Department of Work Psychology and Physiology, Gydas vei 8, Oslo, Norway; Curtin University, AUSTRALIA

## Abstract

**Objectives:**

The aim was to determine the association of occupational arm inclination with shoulder pain in construction and health care workers.

**Methods:**

Arm inclination relative to the vertical was measured with an accelerometer placed on the dominant upper arm for up to four full days at baseline in 62 construction workers and 63 health care workers. The pain intensity in the shoulder and mechanical and psychosocial work factors were measured by self-reports at baseline and prospectively after 6 months. The associations between exposures and shoulder pain were analyzed with multilevel mixed-effects linear regressions.

**Results:**

For the total study population working with the dominant arm at inclinations > 30° and >120° was associated with lower levels of shoulder pain both cross-sectionally and after 6 months. Associations were attenuated when adjusting for individual and social factors, psychological state, and exposure during leisure time, especially for the high inclination levels. Analyses, only including subjects with no pain at baseline revealed no significant associations. While stratified analysis showed negative associations in the construction worker group, there were no significant association in health care workers. Compared to the number of hypotheses tested, the number of significant findings was low. Adjustment by Bonferroni-correction made almost all findings insignificant.

**Conclusions:**

All analyses reflected a negative association between arm inclination and shoulder pain, but few analyses showed these associations to be statistically significant. If there is a relationship between arm inclination and shoulder pain, these findings could indicate that pain-avoidance may modify how workers perform their tasks.

## Background

Shoulder pain is a major concern in Western society [1]. The one-month prevalence of shoulder pain in the general population ranges from 19% to 31% and the lifetime prevalence from 7% to 67% [1]. The health care [2,3] and construction [4] sectors are known for a high prevalence of shoulder complaints. Knowledge of work-related risk factors for shoulder complaints are needed regarding individual health, work ability, loss of productivity [5], and socio-economic costs [6].

A recent systematic review [7] identified seven high-quality studies with prospective designs based on subjective reports of mechanical exposures at work, that all found positive associations between work with hands above shoulder level and shoulder pain [8–14]. Despite the fact, that all these associations were insignificant, the authors of the review concluded: “The evidence is strong that there is an association between shoulder disorders and working with hands above shoulder level” [7]. Self-reports of some mechanical exposures at work have inadequate validity. The duration of exposure is often overestimated [15,16]. Correlations between objectively measured data and self-reports are relatively low [17]. Bias in self-reported measurements might be caused by recall bias or insufficient memory, interpretation of questions, pain [17–19], perception of other work factors, or psychosocial factors [20]. Therefore, objective measurements of exposures are recommended to provide valid assessments of exposure [18].

We have only found three studies of mechanical exposures in association with shoulder pain based on objective measurements. Punnett et al. [21] found, based on video recording of automobile workers, an *increased risk of shoulder pain* when working with severe shoulder flexion or abduction especially for more than 10% of the work cycle. A cross-sectional study by Svendsen and co-workers [22] reported *higher odds ratios for various shoulder disorders* with the increased duration of work with upper arm elevation above 90°. These two studies pertained to middle-aged workers, mostly men, in industrial settings. Hanvold et al. [23] followed young workers from school and during their early working life. They found an association between the duration of arm inclination above 60° and 90° and shoulder pain only to be significant among women [23]. Hence, there is a lack of knowledge of effects of work with elevated arms on shoulder complaints and disorders.

The aim of the present study was to determine whether objectively measured arm inclination at work is associated with shoulder pain in construction and health care workers cross-sectional and prospectively. In addition, we aimed to determine the effect size of various exposure ranges (duration of arm inclination above various levels) on shoulder pain.

## Methods

### Study population and design

This study was part of a larger prospective cohort study described previously by Lunde and co-workers [24]. The subjects for this study were recruited from four construction companies and two local health care providers in the area of Oslo from April 2014 to May 2015. At baseline, 594 participants (construction workers: n = 293, response rate 50.5%; health care workers: n = 301, response rate 51.4%) responded to a questionnaire. One hundred seventy-eight responders in construction work and 193 responders in health care work consented to participate in the technical measurements, and 125 workers were selected for technical measurements based on logistics (availability, work schedules and occupational titles). A detailed overview of the included professions was previously reported by Koch et.al. [15]. Eighty-six participants among those with technical measurements reported concerning shoulder complaints in a questionnaire 6 months later (see Table 1).

**Table 1:** Descriptive statistics of the study population.

	n					
Technical measurements examined	125					
Technical measurements with valid data	113					
Responders to 6-month questionnaire	86					
Construction workers	56					
Health care workers	57					
	Total		Construction work		Health care work	
	Mean	SD	Mean	SD	Mean	SD
Individual factors:						
Age [years]*	42.5	11.8	39.8	13.3	45.1	9.4
Height [cm]	173.6	9.5	179.8	6.5	167.6	8.1
Weight [kg]	76.5	13.2	82.4	11.7	70.5	12.1
BMI [kg/m^2^]	25.3	3.6	25.5	3.4	25.1	3.8
Work exposure [% of time]:						
Arm inclination >30°	36.7	12.1	40.6	13.1	32.8	9.8
Arm inclination >60°	7.0	6.2	9.0	6.7	5.0	5.0
Arm inclination >90°	2.1	3.5	2.7	3.4	1.4	3.6
Arm inclination >120°	0.4	0.6	0.6	0.7	0.2	0.2
Leisure exposure [% of time]:						
Arm inclination >30°	44.5	14.3	47.3	13.2	41.9	14.8
Arm inclination >60°	10.3	7.7	12.7	9.0	8.1	5.4
Arm inclination >90°	2.5	3.1	3.1	3.5	1.9	2.6
Arm inclination >120°	1.0	1.6	1.5	2.2	0.5	0.6
Shoulder pain at baseline [0–3]**						
Dominant shoulder	0.7	0.9	0.5	0.8	0.9	0.9
Opposite shoulder	0.5	0.7	0.3	0.6	0.6	0.8
Shoulder pain at 6 months [0–3]**						
Dominant shoulder	0.7	0.9	0.5	0.7	0.9	0.9
Opposite shoulder	0.4	0.8	0.4	0.8	0.5	0.8
Psychological state [0–3]**						
Psychological complaint severity index(PSI)	0.3	0.5	0.3	0.5	0.4	0.5
Psychological and organizational factors [0–3]***						
Quantitative job demands	3.0	0.7	3.0	0.6	3.1	0.7
Decision control during work	3.1	0.7	3.2	0.7	3.0	0.8
Pacing control	2.9	0.7	2.9	0.6	2.9	0.8
Social climate in the organization	3.2	0.5	3.1	0.5	3.2	0.6
Self-reported work with hands above shoulder height [0–5]****						
Baseline	1.0	1.2	1.2	1.3	0.9	1.2
6-months	1.7	1.0	1.9	1.1	1.5	0.9

* Range [y]: 19–67 (Total); 19–67 (Construction work); 20–64 (Health care work)

** Response alternatives: (0) not troubled, (1) little troubled, (2) partly troubled, and (3) seriously troubled

*** Response alternatives: (0) never, (1) rather little, (2) somewhat, (3) rather much, and (4) very much

**** Response alternatives: (0) never, (1) sometimes, (2) approximately 25% of the time, (3) approximately 55% of the time, (4) approximately 75% of the time, and (5) all the time

Exclusion criteria for the study were inadequate skills in reading and writing Norwegian; a diagnosis of cardiovascular disease or known allergic reaction to plaster, tape, and bandages; or being pregnant. All participants had a physical examination by a physician or a nurse one day prior to the start of the technical measurements.

### Ethical aspects

Prior to participation, all subjects were informed of the purpose and methods of the study and signed a written consent form. This study was conducted in accordance with the 1964 Helsinki Declaration and was approved by the Regional Committee for Medical and Health Research Ethics in Norway (2014/138/REK sør-øst D).

### Arm inclination

Arm inclination relative to the vertical (including both anteversion/retroversion and abduction/adduction) was measured on the dominant arm of the participant during up to four consecutive days using an ActiGraph GT3X+ sensor (ActiGraph LLC, Pensacola, Florida, United States; sampling frequency: 30 Hz). The sensor was attached to the skin 3 cm below the deltoid muscle insertion on the humerus using double-sided tape (Fixomull; BSN Medical, Hamburg, Germany). The accelerometer was covered with transparent film (Tegaderm; 3M, St. Paul, Minnesota, United States) for better fixation. The Actigraph GT3X+ sensors were found to be valid for measuring the inclination of the upper arm and body [25]. For each day, the participants were asked to write down the time of start and stop of each working, leisure and sleep period.

The raw data from the Actigraph GT3X+ sensor were stored on a personal computer using Actilife 6.11.5 software (Actigraph LLC, Pensacola, Florida). The mean durations (minutes) in the arm positions above 30, 60, 90, and 120° for an individual’s working and leisure periods were calculated based on the raw data of the measurements and the participants’ diaries with custom-made software Acti4 [25,26]. The data were excluded when the sensor was removed from the arm, and the working period was shorter than four hours or 75% of the mean average length of all working periods. Due to these exclusion criteria and the fact that working schedules often were shorter on Fridays, a data collection duration of three to four days (Monday to Wednesday/Thursday) was most practicable. *Compared to single day measurements*, *repeated measures for several working days are recommend to increase reliability* [15].

For each variable of the arm inclination (above 30, 60, 90, and 120°), the mean value as a percentage of time for the working periods of all measuring days for each person was calculated.

### Shoulder pain

The intensity of the shoulder pain of both arms was rated on a four-point scale (0 = not troubled, 1 = little troubled, 2 = partly troubled and 3 = seriously troubled) [27] at baseline and after 6 months. Only pain intensity in the shoulder of the participant’s dominant arm was included in this study.

### Individual factors

Individual factors such as advanced age, female gender, and a high body mass index (BMI) have been shown in previous studies to increase the risk of shoulder pain [28,29] and were assessed by questionnaire at the baseline of this study.

### Psychological/social factors and psychological state

Psychological and social factors [30,31] have been reported as work-related risk factors for shoulder pain. In this study, psychological and social factors were measured by 30 questions of the General Nordic Questionnaire (QPS_Nordic_) [32]. Quantitative job demands (four items), control of decisions (five items), control of work pacing (four items), and social climate (three items) were calculated by the mean of the corresponding single items (see appendix).

Psychological complaints (fear, depression, fatigue) were rated on a four-point scale for intensity (0 = not troubled, 1 = little troubled, 2 = partly troubled and 3 = seriously troubled). A psychological complaint severity index (PSI) was calculated as the mean of all single complaints [27].

### Self-reported arm inclination

Work with inclined arms was screened by self-reports at baseline and after 6 months by the following question: “How often in your daily work are you exposed to work with hands above shoulder height?” The answer categories were “never” (0), “sometimes” (1), “approximately 25% of the time” (2), “approximately 50% of the time” (3), “approximately 75% of time” (4), and “all the time” (5).

### Statistical analyses

The association between arm inclination and shoulder pain, both at baseline and after 6 months, was investigated using multilevel mixed-effects linear regression fitted via restricted maximum likelihood. We analyzed the duration of working time (percentage of workday) spent at four different levels of arm angle: >30°, >60°, >90°, and >120°. For each of the four levels we implemented the following models: (1) Shoulder pain (at baseline and after 6 months) was entered as the dependent variable and duration of arm inclination at the respective angle as covariate. An interaction term between time and arm inclination enabled us to study the association between should pain and arm inclination at baseline and after 6 month, respectively; (2) Model 1 adding individual factors of age, sex, BMI and work sector (construction and health care); (3) Model 2 adding self-reported psychological and social factors (social climate, quantitative job demands, decision control at work, and pacing control at work); and (4) Model 3 adding the psychological complaint severity index adding and arm inclination during leisure time. All variables were selected prior to analyses. We treated gender and working sectors as categorical variables. Analyses were performed for the total study population and stratified by work sector (construction and health care).

We performed linear regressions for Model 1 to Model 4 between work exposure and shoulder pain after 6 month, excluding all participants reporting pain at baseline.

To elucidate the effect of the percentage of working time with arm inclination >30° and >120° on shoulder pain, generalized additive mixed models (GAMMs) were performed for both time points. All confounding variables were included. Regression splines were implemented with 4 degrees of freedom and represented the effect of arm inclination on pain. Depending on the effect patterns, regression coefficients were calculated for selected exposure ranges.

To determine the changes in arm exposure between the baseline and 6 months, we performed Wilcoxon rank tests on self-reported arm exposure. We assumed that the expected overestimation of the exposure duration [15] [16] was equal at both time points.

*Supplementary analyses—*For sensitivity testing, the association between arm inclination during work and shoulder pain was examined using linear mixed regressions with the exposure variable absolute duration of arm inclination in minutes. Furthermore, the percentage full day exposure (Sum of work- and leisure time) with arm inclination above various levels was associated to shoulder pain. For linear mixed models with full-day exposure, only models 1 to 4 were performed.

Statistical data analyses were performed with IBM SPSS Statistics 23 (IBM Corporation, New York, United States), STATA (version 13.0, StataCorp. College Station, TX, USA) and the Mixed GAM Computation Vehicle package (version 1.8–14) in R (https://www.r-project.org).

## Results

While the variables age, BMI and arm inclination above 30° during work and leisure were normally distributed, the other arm inclination variables were not.

For the total study population the percentage of time spent with arm inclination above 30°, 60°, 90° and 120° was 36.7% (SD: 12.1%), 7.0% (SD: 6.2%), 2.1% (SD: 3.5%) and 0.4% (SD: 0.6%) during work and 44.5% (SD: 14.3%), 10.3% (SD: 7.7%), 2.5% (SD: 3.1%), and 1.0% (SD: 1.6%) during leisure (see Table 1). We found no significant changes in self-reported work with the hands above shoulder height between baseline and at 6 months; therefore, we assumed the exposure with inclined arms to be unchanged between these two time points.

Sixty (53.1%) participants reported no pain at baseline, and 47 (41.6%) reported no pain at 6 months. Thirty participants reported pain both at baseline and after 6 months. In total, the mean values for pain intensity were 0.8 (SD: 0.9) at baseline and 0.7 (SD: 0.9) after 6 months.

Significant differences between responders and non-responders of the 6-month questionnaire, were found for gender (non-responders: 21 men, 6 women, responders: 47 men, 39 women) and pacing control at work (non-responders: 2.6 (SD: 0.8), responders: 3.0 (SD. 0.7)).

### Association of arm inclination during work with shoulder pain

Significant associations with shoulder pain were found in the crude models for the *percentage of working time with arm inclination* >30° and >120°, both at baseline (>30°: β = -0.02, CI = -0.03 to -0.00; >120°: β = -0.45, CI = -0.71 to -0.19) and at 6 months (>30°: β = -0.02, CI = -0.03 to 0.00; >120°: β = -0.37, CI = -0.64 to -0.10) (see Table 2). Additionally, we found significant associations between arm inclination >60° and shoulder pain at 6 months (β = -0.02, CI = -0.05 to -0.00). When adjusting for covariates (Model 2 to Model 5), the association of arm inclination >30° and shoulder pain at baseline and at 6 months remained significant. For arm inclination >120°, the association with shoulder pain at baseline was maintained in all models; however, for shoulder pain at 6 months, significant associations were found only when adjusting for individual factors, psychological and social factors (Models 2 and 3). In all analyses, the β-coefficients of the association of arm inclination and shoulder pain at both time points were negative.

**Table 2:** Linear mixed models with arm-inclination exposure at work [% of total time at work] and shoulder pain.

	Time		Model 1			Model 2			Model 3			Model 4	
		n/obs. = 113/199			n/obs. = 111/195			n/obs. = 109/191			n/obs. = 97/169		
		β	95% CI	P	β	95% CI	P	β	95% CI	P	β	95% CI	P
Arm inclination >30°	T1	-0.02	-0.03,-0.00	**0.012**	-0.02	-0.03,-0.00	**0.018**	-0.02	-0.03,-0.00	**0.021**	-0.02	-0.03,-0.00	**0.041**
	T2	-0.02	-0.03,-0.00	**0.009**	-0.02	-0.03,-0.00	**0.014**	-0.02	-0.03,-0.00	**0.018**	-0.02	-0.03,-0.00	**0.027**
Arm inclination >60°	T1	-0.02	-0.05,0.00	0.063	-0.02	-0.04,0.01	0.150	-0.02	-0.05,0.00	0.105	-0.02	-0.05,0.01	0.278
	T2	-0.02	-0.05,-0.00	**0.048**	-0.02	-0.05,0.00	0.110	-0.02	-0.05,0.01	0.123	-0.02	-0.05,0.01	0.286
Arm inclination >90°	T1	-0.03	-0.07,0.01	0.187	-0.02	-0.07,0.02	0.268	-0.05	-0.11,0.01	0.103	-0.04	-0.11,0.02	0.206
	T2	-0.04	-0.09,0.00	0.077	-0.04	-0.08,0.01	0.108	-0.05	-0.11,0.02	0.149	-0.04	-0.11,0.03	0.251
Arm inclination >120°	T1	-0.45	-0.71,-0.19	0.001*	-0.39	-0.67,-0.11	**0.007**	-0.43	-0.71,-0.14	**0.004**	-0.31	-0.61,-0.01	**0.044**
	T2	-0.37	-0.64,-0.10	**0.007**	-0.32	-0.61,-0.03	**0.032**	-0.34	-0.64,-0.05	**0.024**	-0.22	-0.53,0.09	0.161

Dependent variables: Pain (T1, T2), continuous (0:3)

Independent variables:

Model 1: Arm inclination work

Model 2: Arm inclination work, Age, BMI, Gender, Working sector

Model 3: Arm inclination work, Age, BMI, Gender, Working sector, Social climate, Quantitative job demands, Decision control, Pacing control

Model 4: Arm inclination work, Age, BMI, Gender, Working sector, Social climate, Quantitative job demands, Decision control, Pacing control, PSI, Arm inclination leisure

* P < 0.002 (adjusted α–level after Bonferroni correction)

By adjusting the α-level to 0.002 due Bonferroni-correction (α_cor_ = 0.05 / 32), only the association of arm inclination >120° and shoulder pain at baseline in Model 1 remained significant.

#### Arm inclination at work and pain after 6 months in baseline-pain free participants

Participants free of shoulder pain at baseline showed no significant associations between work-arm inclination and shoulder pain (see Table 3).

**Table 3:** Linear regression models with arm-inclination exposure at work [% of total time at work] and shoulder pain (excluded participants reporting pain at baseline).

		Model 1			Model 2			Model 3			Model 4		
			n = 86			n = 84			n = 82			n = 73	
	Time	β	95% CI	P	β	95% CI	P	β	95% CI	P	β	95% CI	P
Arm inclination >30° [%]	T2	0.01	-0.05,0.06	0.827	0.01	-0.06,0.07	0.814	-0.01	-0.08,0.07	0.861	-0.01	-0.08,0.07	0.853
Arm inclination >60° [%]	T2	0.00	-0.09,0.09	0.945	-0.00	-0.10,0.10	0.977	-0.03	-0.15,0.09	0.585	-0.03	-0.15,0.09	0.601
Arm inclination >90° [%]	T2	-0.07	-0.34,0.21	0.628	-0.09	-0.40,0.21	0.551	-0.23	-0.66,0.19	0.283	-0.23	-0.66,0.19	0.287
Arm inclination >120° [%]	T2	0.12	-0.82,1.05	0.803	0.20	-0.92,1.33	0.723	-0.05	-1.33,1.23	0.937	-0.03	-1.30,1.24	0.961

T2: 6 month

Dependent variable: Pain T2, continuous (0:3)

Independent variables

Model 1: Arm inclination work

Model 2: Arm inclination work, Age, BMI, Gender, Working sector

Model 3: Arm inclination work, Age, BMI, Gender, Working sector, Social climate, Quantitative job demands, Decision control, Pacing control

Model 4: Arm inclination work, Age, BMI, Gender, Working sector, Social climate, Quantitative job demands, Decision control, Pacing control, PSI, arm inclination leisure

#### Work sector: Stratified analyses

For construction workers, we found significant associations in the crude analyses of arm inclination >30° and >120° and shoulder pain at baseline (>30°: β = -0.01, CI = -0.03 to 0.00; >120°: β = -0.37, CI = -0.63 to 0.11 and at 6 months (>30°: β = -0.01, CI = -0.03 to 0.00; >120°: β = -0.28, CI = -0.54 to 0.01) (see appendix, Table 4). For both arm inclination >30° and >60°, the associations with shoulder pain at baseline and after 6 months were significant/close to significant when adjusting for individual factors, psychological and organizational factors, as well as psychological state (Models 2–4). All β-coefficients in the analyses were negative.

**Table 4:** Linear mixed model with arm inclination exposure at work [% of total time at work] and shoulder pain: stratified analyses for construction and health care work*.

		**Model 1**			**Model 2**			**Model 3**			**Model 4**		
**Construction work**		n/obs. = 56/97			n/obs. = 56/97			n/obs. = 56/97			n/obs. = 49/84		
		β	95% CI	P	β	95% CI	P	β	95% CI	Sig.	β	95% CI	P
Arm inclination >30°	T1	-0.01	-0.03,-0.00	**0.047**	-0.01	-0.03,0.00	0.053	-0.01	-0.03,-0.00	**0.048**	-0.02	-0.04,0.00	0.107
	T2	-0.01	-0.03,-0.00	**0.045**	-0.01	-0.03,-0.00	**0.048**	-0.01	-0.03,-0.00	**0.044**	-0.02	-0.04,0.00	0.110
Arm inclination >60°	T1	-0.02	-0.05,0.01	0.182	-0.02	-0.05,0.01	0.238	-0.02	-0.05,0.01	0.201	-0.01	-0.05,0.02	0.466
	T2	-0.02	-0.05,0.01	0.232	-0.02	-0.05,0.01	0.283	-0.02	-0.05,0.01	0.239	-0.01	-0.05,0.03	0.559
Arm inclination >90°	T1	-0.04	-0.10,0.02	0.170	-0.04	-0.10,0.02	0.203	-0.04	-0.10,0.02	0.184	-0.04	-0.11,0.04	0.356
	T2	-0.03	-0.09,0.02	0.241	-0.03	-0.09,0.03	0.270	-0.04	-0.10,0.03	0.244	-0.03	-0.11,0.05	0.434
Arm inclination >120°	T1	-0.37	-0.63,-0.11	**0.005**	-0.37	-0.62,-0.11	**0.005**	-0.37	-0.64,-0.10	**0.008**	-0.37	-0.69,-0.04	**0.027**
	T2	-0.28	-0.54,-0.01	**0.043**	-0.28	-0.54,-0.02	**0.037**	-0.28	-0.56,-0.00	0.050	-0.27	-0.60,0.07	0.118
		**Model 1**			**Model 2**			**Model 3**			**Model 4**		
**Health care work**		n/obs = 57/102			n/obs = 55/98			n/obs = 53/94			n/obs = 48/85		
		β.	95% CI	P	β	95% CI	P	β	95% CI	P	β	95% CI	P
Arm inclination >30°	T1	-0.01	-0.03,0.01	0.459	-0.02	-0.04,0.01	0.236	-0.02	-0.05,0.01	0.157	-0.03	-0.05,-0.00	**0.031**
	T2	-0.01	-0.03,0.01	0.388	-0.02	-0.04,0.01	0.203	-0.02	-0.05,0.01	0.141	-0.03	-0.06,-0.01	**0.015**
Arm inclination >60°	T1	-0.01	-0.05,0.04	0.731	-0.01	-0.06,0.04	0.614	-0.03	-0.08,0.03	0.376	-0.03	-0.09,0.03	0.326
	T2	-0.02	-0.07,0.03	0.412	-0.02	-0.07,0.02	0.332	-0.03	-0.09,0.03	0.335	-0.04	-0.10,0.02	0.191
Arm inclination >90°	T1	-0.00	-0.07,0.07	0.963	-0.00	-0.07,0.06	0.897	-0.06	-0.23,0.12	0.510	-0.12	-0.31,0.06	0.187
	T2	-0.03	-0.10,0.04	0.354	-0.03	-0.10,0.03	0.323	-0.05	-0.23,0.12	0.547	-0.13	-0.32,0.05	0.152
Arm inclination >120°	T1	-0.47	-1.47,0.54	0.365	-0.53	-1.57,0.51	0.316	-0.38	-1.48,0.72	0.499	-0.34	-1.50,0.82	0.566
	T2	-0.58	-1.67,0.50	0.293	-0.68	-1.78,0.42	0.224	-0.36	-1.54,0.83	0.555	-0.38	-1.60,0.84	0.541

T1: baseline; T2: 6 month

Dependent variables: Pain (T1, T2), continuous (0:3)

Independent variables:

Model 1: Arm inclination work

Model 2: Arm inclination work, Age, BMI, Gender, Working sector

Model 3: Arm inclination work, Age, BMI, Gender, Working sector, Social climate, Quantitative job demands, Decision control, Pacing control

Model 4: Arm inclination work, Age, BMI, Gender, Working sector, Social climate, Quantitative job demands, Decision control, Pacing control, PSI, Arm inclination leisure

* no significant associations were found after adjustment of α–level by Bonferroni correction (α_cor_ = 0.002)

Health care workers showed a significant association between arm inclination >30° and shoulder pain at baseline (β = -0.03, CI = -0.05 to 0.00) and after 6 months (β = -0.03, CI = -0.06 to 0.00) when adjusting for all covariates (Model 5). Analyses of the associations between arm inclination and shoulder pain for health care workers showed highly significant associations of confounding PSI on shoulder pain (Models 4 + 5; β, CIs and p-values for PSI not included in the tables).

No significant associations could be found for neither the construction- nor the health care sector when adjusting the α-level by Bonferroni-correction.

#### Supplementary analyses

The associations of shoulder pain and absolute duration of arm inclination above various levels measured in minutes were similar to those found with the arm inclination measured as the percentage of work time. In crude analyses, significant associations were found for the same combinations of various levels of arm inclination and shoulder pain at baseline and at 6 months. In the adjusted analyses (Model 2 to Model 5), no significant changes were found for arm inclination >30° compared with analyses with exposure variables measured as percentages (see appendix, Table 5).

**Table 5:** Linear mixed model with absolute duration of arm inclination exposure (minutes) and shoulder pain*.

		Model 1			Model 2			Model 3			Model 4		
		n/obs. = 113/199			n/obs. = 111/195			n/obs. = 109/191			n/obs. = 97/169		
		β	95% CI	P	β	95% CI	P	β	95% CI	P	β	95% CI	P
Arm inclination >30°	T1	-0.00	-0.00,-0.00	**0,04**	-0.00	-0.00,0.00	0,11	-0.00	-0.00,0.00	0,11	-0.00	-0.00,0.00	0,36
	T2	-0.00	-0.01,-0.00	**0,03**	-0.00	-0.01,0.00	0,08	-0.00	-0.01,0.00	0,08	-0.00	-0.00,0.00	0,25
Arm inclination >60°	T1	-0.01	-0.01,0.00	0,06	-0.00	-0.01,0.00	0,20	-0.00	-0.01,0.00	0,16	-0.00	-0.01,0.00	0,46
	T2	-0.01	-0.01,-0.00	**0,04**	-0.00	-0.01,0.00	0,13	-0.01	-0.01,0.00	0,15	-0.00	-0.01,0.00	0,41
Arm inclination >90°	T1	-0.01	-0.02,0.00	0,16	-0.01	-0.02,0.00	0,28	-0.01	-0.03,0.00	0,13	-0.01	-0.03,0.01	0,23
	T2	-0.01	-0.02,0.00	0,07	-0.01	-0.02,0.00	0,12	-0.01	-0.02,0.00	0,17	-0.01	-0.02,0.01	0,26
Arm inclination >120°	T1	-0.09	-0.15,-0.04	**0,00**	-0.08	-0.14,-0.02	**0,01**	-0.09	-0.15,-0.03	**0,01**	-0.06	-0.13,0.00	0,05
	T2	-0.07	-0.13,-0.02	**0,01**	-0.06	-0.12,-0.00	**0,05**	-0.07	-0.13,-0.01	**0,03**	-0.04	-0.11,0.02	0,18

T1: baseline; T2: 6 month

Dependent variables: Pain (T1, T2), continuous (0:3)

Independent variables:

Model 1: Arm inclination work

Model 2: Arm inclination work, Age, BMI, Gender, Working sector

Model 3: Arm inclination work, Age, BMI, Gender, Working sector, Social climate, Quantitative job demands, Decision control, Pacing control

Model 4: Arm inclination work, Age, BMI, Gender, Working sector, Social climate, Quantitative job demands, Decision control, Pacing control, PSI, Arm inclination leisure

* no significant associations were found after adjustment of α–level by Bonferroni correction (α_cor_ = 0.002)

Analyses with full-day exposure (percentage of time and absolute duration in minutes) showed similar results for the crude analyses of shoulder pain and arm inclination to analyses performed with work exposures. For the adjusted models, fewer significant associations were found; however, all β-coefficients remained negative (see appendix, Table 6 and Table 7).

**Table 6:** Linear mixed model with full day arm-inclination exposure (percentage of 24 hours) and shoulder pain*.

			Model 1			Model 2			Model 3			Model 4	
		n/obs. = 102/178			n/obs. = 101/176			n/obs. = 99/172			n/obs. = 97/169		
		β	95% CI	P	β	95% CI	P	β	95% CI	P	β	95% CI	P
Arm inclination >30°	T1	-0.01	-0.03,0.00	0,05	-0.01	-0.03,0.00	0,10	-0.01	-0.03,0.00	0,09	-0.01	-0.03,0.00	0,09
	T2	-0.01	-0.03,-0.00	**0,04**	-0.01	-0.03,0.00	0,08	-0.01	-0.03,0.00	0,07	-0.01	-0.03,0.00	0,07
Arm inclination >60°	T1	-0.03	-0.05,-0.00	**0,04**	-0.02	-0.05,0.01	0,16	-0.03	-0.06,-0.00	**0,04**	-0.03	-0.06,0.00	0,05
	T2	-0.03	-0.06,-0.00	**0,04**	-0.02	-0.05,0.01	0,15	-0.03	-0.06,-0.00	**0,05**	-0.03	-0.06,0.00	0,06
Arm inclination >90°	T1	-0.04	-0.10,0.01	0,14	-0.03	-0.08,0.03	0,33	-0.08	-0.16,-0.00	**0,05**	-0.06	-0.14,0.01	0,08
	T2	-0.05	-0.10,0.01	0,12	-0.03	-0.09,0.02	0,26	-0.06	-0.14,0.02	0,12	-0.05	-0.13,0.02	0,19
Arm inclination >120°	T1	-0.20	-0.35,-0.04	**0,01**	-0.15	-0.32,0.02	0,08	-0.16	-0.32,0.01	0,06	-0.16*	-0.31,-0.00	**0,05**
	T2	-0.17	-0.35,0.00	0,05	-0.13	-0.31,0.06	0,18	-0.14	-0.32,0.05	0,14	-0.14	-0.31,0.03	0,12

T1: baseline; T2: 6 month

Dependent variables: Pain (T1, T2), continuous (0:3)

Independent variables:

Model 1: Arm inclination work + leisure

Model 2: Arm inclination work + leisure, Age, BMI, Gender, Working sector

Model 3: Arm inclination work + leisure, Age, BMI, Gender, Working sector, Social climate, Quantitative job demands, Decision control, Pacing control

Model 4: Arm inclination work + leisure, Age, BMI, Gender, Working sector, Social climate, Quantitative job demands, Decision control, Pacing control, PSI

* no significant associations were found after adjustment of α–level by Bonferroni correction (α_cor_ = 0.002)

**Table 7:** Linear mixed model with full day absolute duration of arm inclination exposure (minutes) and shoulder pain*.

			Model 1			Model 2			Model 3			Model 4	
		n/obs. = 102/178			n/obs. = 101/176			n/obs. = 99/172			n/obs. = 97/169		
		β	95% CI	P	β	95% CI	P	β	95% CI	P	β	95% CI	P
Arm inclination >30°	T1	-0.00	-0.00,-0.00	**0,02**	-0.00	-0.00,0.00	0,08	-0.00	-0.00,0.00	0,06	-0.00	-0.00,0.00	0,07
	T2	-0.00	-0.00,-0.00	**0,01**	-0.00	-0.00,0.00	0,06	-0.00	-0.00,-0.00	**0,05**	-0.00	-0.00,-0.00	**0,05**
Arm inclination >60°	T1	-0.00	-0.01,-0.00	**0,03**	-0.00	-0.00,0.00	0,17	-0.00	-0.01,-0.00	**0,04**	-0.00	-0.01,0.00	0,05
	T2	-0.00	-0.01,-0.00	**0,03**	-0.00	-0.00,0.00	0,15	-0.00	-0.01,-0.00	**0,05**	-0.00	-0.01,0.00	0,05
Arm inclination >90°	T1	-0.00	-0.01,0.00	0,12	-0.00	-0.01,0.00	0,33	-0.01	-0.02,-0.00	**0,05**	-0.01	-0.01,0.00	0,09
	T2	-0.00	-0.01,0.00	0,11	-0.00	-0.01,0.00	0,29	-0.01	-0.01,0.00	0,12	-0.01	-0.01,0.00	0,18
Arm inclination >120°	T1	-0.02	-0.04,-0.00	**0,01**	-0.01	-0.03,0.00	0,08	-0.02	-0.03,0.00	0,06	-0.02	-0.03,-0.00	**0,05**
	T2	-0.02	-0.04,0.00	0,05	-0.01	-0.03,0.01	0,19	-0.01	-0.03,0.00	0,14	-0.01	-0.03,0.00	0,11

T1: baseline; T2: 6 month

Dependent variables: Pain (T1, T2), continuous (0:3)

Independent variables:

Model 1: Arm inclination work + leisure

Model 2: Arm inclination work + leisure, Age, BMI, Gender, Working sector

Model 3: Arm inclination work + leisure, Age, BMI, Gender, Working sector, Social climate, Quantitative job demands, Decision control, Pacing control

Model 4: Arm inclination work + leisure, Age, BMI, Gender, Working sector, Social climate, Quantitative job demands, Decision control, Pacing control, PSI

* no significant associations were found after adjustment of α–level by Bonferroni correction (αcor = 0.002)

### Duration of arm inclination and shoulder pain

**Fig 1:**
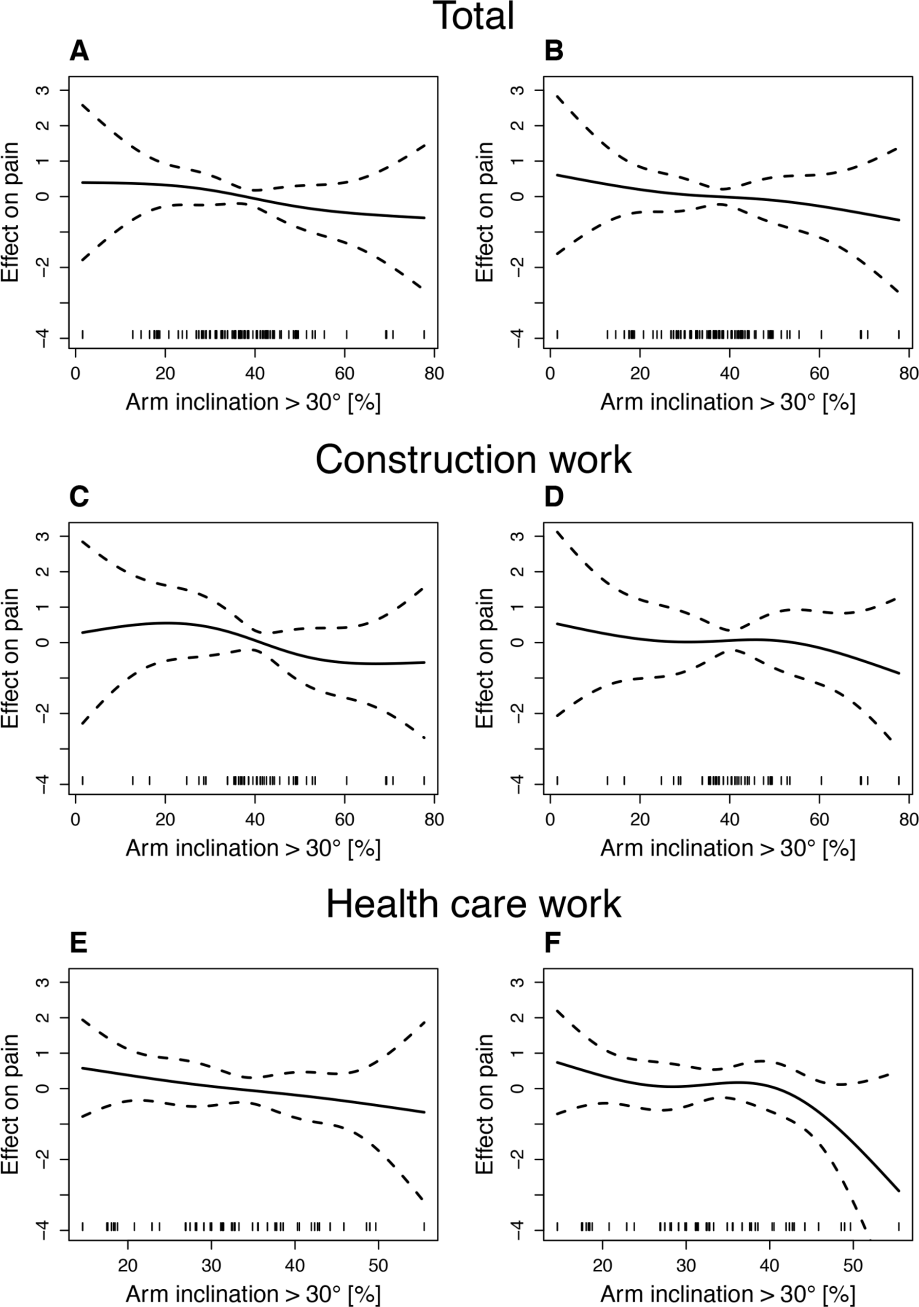
Estimated splines for the effect of arm inclination >30° on shoulder pain. A: Total group at baseline. B: Total group at 6 months. C: Construction work at baseline. D: Construction work at 6 months. E: Health care work at baseline. F: Health care work at 6 months. The black solid lines represent the estimated splines for arm inclination effects on pain levels from GAM models, and the black dashed lines represent 95% confidence bands.

**Fig 2:**
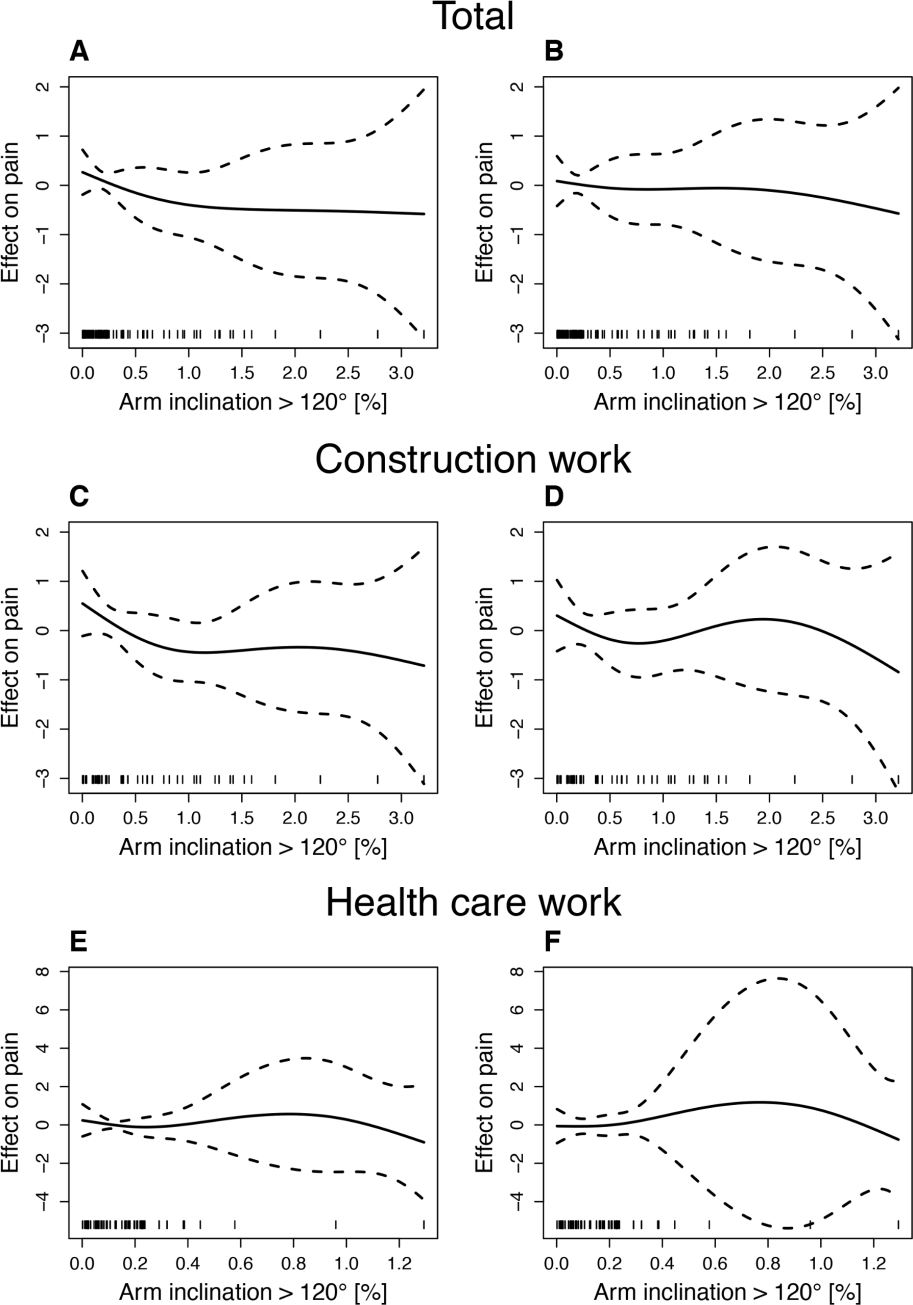
Estimated splines for the effect of arm inclination >120° on shoulder pain. A: Total group at baseline. B: Total group at 6 months. C: Construction work at baseline. D: Construction work at 6 months. E: Health care work at baseline. F: Health care work at 6 months. Black solid lines represent the estimated splines for arm inclination effects on pain levels from GAM models, and the black dashed lines represent 95% confidence bands.

Estimations of the effect on shoulder pain were adjusted for all covariates and were performed for arm inclination >30° and >120° based on the results of linear mixed regressions. The effect curves for arm inclination >30° are displayed in Fig 1 and those for arm inclination >120 in Fig 2.

#### Effect of work with arm inclination >30°

For the total group, the estimated splines of the effect of work with arm inclination >30° on shoulder pain both at baseline and at 6 months showed an almost linear decreasing pattern with increasing exposure duration (see Fig 1A and 1B). In the range from 20% to 50% of the working time, the slopes were -0.021 (baseline) and -0.010 (6 months). The corresponding risk ratio (RR) for the change of 10% of working time pain was -0.2 units of shoulder pain at baseline and -0.1 units of shoulder pain at 6 months.

Construction workers were found to exhibit bimodal patterns of the effect of arm inclination on baseline pain and shoulder pain after 6 months (see Fig 1C and 1D) with piecewise linearity between 30% and 50% of the working time. While in the range of 30% to 50% for baseline pain, a negative slope (β = -0.039) was observed, and the slope for shoulder pain after 6 months was positive (β = 0.002).

For health care workers, the effect of arm inclination on baseline shoulder pain showed an almost linear descending slope (β = -0.030) over the whole range of the percentage of the working time. For shoulder pain at 6 months, the estimated curve exhibited a wave-like pattern with an initial minimum at approximately 27% of the working time and a strong decreasing slope from 47% of the working time.

#### Effect of work with arm inclination >120°

The effect of arm inclination >120° on baseline shoulder pain in the total group was found to continuously decrease from 0% to 0.5% of the working time (β = -0.837). From 0.5%, the rise of the effect pattern became lower, passing into a piecewise linear decreasing trend from 1.5% to 3.0% (β = -0.054). The effect of arm inclination on 6-month shoulder pain in the total group slightly decreased (β = -0.280) from 0% to 0.5%, followed by a part with almost no effect until 1.5% and a negatively increasing effect from 1.5%.

For construction workers, a piecewise linear effect was found in the estimated curves from 0 to 0.5% of the working time. The effect on shoulder pain at baseline and at 6 months had a value of β = -1.348 and β = -0.967, respectively. For both baseline and 6-month shoulder pain, the estimated curves showed over 0.5% of a wave-like pattern with a maximum at approximately 2.2% of the working time.

Health care workers’ estimated effect curve of arm inclination on baseline shoulder pain showed a descending pattern from 0% to 0.2% of the working time (β = -1.711). From 0.3% to 0.6%, an almost linear increasing effect (β = 1.665) was found. It should be noted that this effect pattern was based on the data of only a few participants. For 6-month shoulder pain, the effect of arm inclination was slightly increasing (β = 0.252) in the range from 0.0% to 0.2% of the working time.

## Discussion

The present results shows negative association between arm inclination and shoulder pain, almost all insignificant when performing Bonferroni correction. The negative association was found for all inclination levels and for both pain reports at baseline and after 6 months. The duration of the dominant arm inclination >30° and >120° were negatively associated with shoulder pain at baseline and after 6 months when adjusting for age, gender, BMI, and psychological and social work factors (Table 2). Adjusting for psychological state and arm inclination during leisure time attenuated the prospective associations for arm inclination >120°. Supplementary analyses of absolute exposure duration (number of minutes) showed similar results. Furthermore, analyses of the associations between full-day exposure and shoulder pain showed similar results in crude analyses.

Workers of the two sectors exhibited different association between arm inclination and pain. While construction workers exhibited significant negative associations of arm inclination >30° and >120° with shoulder pain, the only statistically significant association in health care workers was for > 30° with adjustment for all covariates (Table 4).

Examining analyses between arm inclination at baseline and shoulder pain after 6 months with participants that did not suffer from pain at baseline we found no significant associations of arm inclination and shoulder pain after 6 months. In contrast to the rest of our findings, some of the calculated regression coefficients were positive, but still were close to zero.

In general, the number of significant associations compared to the number of hypotheses tested was low. When adjusting the α-level by Bonferroni- correction, almost all significant associations disappeared. Nevertheless, all associations in all analyses between arm inclination and shoulder pain were negative.

The finding of a pain-attenuating effect of arm inclination >120° in the present study contrasts with previous findings based on objective measurements [21–23] and with findings based on subjective reports [7–14]. Despite their different designs (cross-sectional, prospective, case-referent), all these studies found a higher risk for shoulder pain with higher exposure durations of arm inclination > 60°. Hence, working with hands over shoulder level was expected to be associated with higher levels of shoulder pain complaints. There are several possible explanations for this discrepancy:

(I) Employees with pain may have learned to avoid postures with high levels of arm inclination as a response to pain (pain-related behavior). Hence, the negative association between arm inclination and pain may be a reverse causation phenomenon: pain may modify ways of coping with exposures at work.

(II) Effects of selection (“healthy-worker effects”). Workers who develop shoulder pain may find it difficult to stay in jobs with high levels of mechanical exposures, hence they move to other types of work. Individuals with shoulder pain due to working with arm inclination may quit these types of jobs.

It might be possible that participants in this study represent a group of workers that suffered less from pain in the past and tolerate more occupational exposures than those who might have already dropped out. In contrast, one of the studies reporting that arm inclination *increased* the risk of shoulder pain [23] investigated young individuals (students entering working life) who were new to the job.

(III) The level of exposure to potentially pathogenic arm inclination was too low to produce pathology in the present study (Table 1). This argument cannot explain the negative associations, however. Compared to previous studies based on objective measurements, durations of arm inclination > 30° were similar to those of construction workers in our study (mean: 41.1%), while the health care workers exhibited somewhat lower numbers (mean: 32.6%). The percentage time spent with arms >30° was 45% (median) in hairdressers, 47% (median) in electricians [23], 32% (mean) in machinists and 39.8% (mean) in painters [22]. The percentage time spent with inclined arms >90° were 2.8% (mean) in construction workers and 1.5% (mean) in health care workers. These numbers were lower than those reported for painters (9.0% mean), car mechanics (4.7% mean) [22], electricians (8% median) [23] and workers performing automobile assembly work (3.9% to 10.0% mean) [21]. However, the present data included the full shift including breaks, which were excluded in the three previous studies.

(IV) Although all associations were in the same direction indicating consistent negative associations between arm inclination and shoulder pain, there were many non-significant test results. Hence, it is possible that there is no physiologically significant association between arm inclination and shoulder pain.

(V) The 6-month follow-up period may be considered short for development of shoulder pathology, but the time factor cannot explain the consistent negative associations between arm inclination and shoulder pain.

However, a recently published study of Coenen et.al. showed similar results as found in our study: arm elevation was negatively associated to shoulder pain at baseline, but not at follow-up [33]. Further studies are necessary to determine the causes of these findings.

Facing the relative low total duration spent with arm inclination >120° the findings might be most important for professions where work with lifted arms is required often. The values in Table 1 represent an average of 113 participants measured over several days. For single days and specific individuals / professions, these values might be clearly higher.

The finding of a pain-attenuating effect of arm inclination >30° seems to indicate that arm movements in general are beneficial to the shoulder. Angles <60° may not present strong biomechanical or physiological challenges to the joint- and tendon-structures of the shoulder joint. However, the present study did not examine movement *patterns* or distributions of duration of arm postures within individuals, hence we cannot determine if movements or arm postures contribute to the observed positive effects on pain.

### Strengths and limitations

The two main strengths of the present study are the objective method used to measure arm inclination and the prospective design. We recorded the arm inclination of 125 participants over several days during work and leisure, achieving a high reliability of exposure measurements [15]. With the inclusion of 16 professions in this study, the variation in daily exposures was high.

The inclusion of individual, psychological and social work factors, and psychological state allowed us to adjust associations with factors previously described in the literature as confounders [7,28].

We analyzed both the percentage of time with arm inclination above various levels as continuous variable and the outcome variable shoulder pain with its four categories in their original scales. To avoid lost of data information, no further subdivision of these variables was made. This provided the full data exploration and did not introduce loss of information about individual differences.

The mixed-effect models are well suited for analyzing inherently unbalanced longitudinal data, consider fixed and random effects, and do not require the same number of observations on each subject. GAM models are flexible and convenient tools to estimate and visualize the effect of a multiple-variable set on an outcome variable based on non-linear associations.

The main limitations were that pain was reported only at two time points (baseline and at 6 months), and arm inclination was measured objectively only at baseline. To examine the long-term effects of arm inclination on shoulder pain, further studies should consider a higher number of time points both with measurements of shoulder complaints and objectively measured exposures. We do not know the pain-history of the study participants, hence we cannot rule out that the study population is selected based on tolerance for working with elevated arms.

We have performed a large number of statistical tests since we chose to test several models (adding one type of factors at each stage) in order to determine if specific factors may confound associations between arm inclination and pain. As mentioned above the number of significant findings compared to the number of hypotheses tested was low. Adjusting the α-levels by Bonferroni corrections would render almost all findings statistically insignificant. However, all effects were in the same direction.

While the results of this study may be generalizable for the two examined working sectors, specific groups of professions can show different associations. In addition, exposures in construction work may vary depending on the construction site, the status of the actual project or seasonal factors. In health care work, exposure variations may be caused by changing patients, patients’ individual disease or behavior, or the number of healthy workers in the working shift of the department [34]. Additionally, psychosocial challenges due to working with patients might be higher in health care work [35].

We found no significant differences in exposure variables and shoulder pain between participants following up the 6-months questionnaire and those who did not. Significant differences were only found in gender and pacing control at work. Dropouts were mostly men and participants with lower control over their working pace. Nevertheless, we believe that these differences have no impact on the generalizability of our findings.

The estimated splines of the effect of arm inclination on shoulder pain in GAM models were affected by the number of data points in specific ranges of the duration of arm inclination. Some ranges included only a few data points, leading to possible bias in the estimations. In stratified analyses, the number of data points was even lower. The results of GAM models would be more consistent when including more participants.

## Conclusion

In contrast to the few previous studies based on objective exposure measurements, the present study found negative associations between arm inclination and shoulder pain in construction workers, but not in health care workers. Despite that almost all significant associations disappeared after Bonferroni-correction, all associations were in the same negative direction. If there is an association between objectively measured arm inclination and shoulder pain, having adapted work behavior patterns to prevent pain may be one explanation of the present findings. Studies of effects of mechanical exposures at work and interventions to prevent pain should take this mechanism into account.

## Appendix

### QPS scales with included single items

Quantitative job demands

1. Is your workload irregular so that the work piles up?
2. Do you have to work overtime?
3. Is it necessary to work at a rapid pace?
4. Do you have too much to do?

Decision control in work

1. If there are alternative methods for doing your work, can you choose which method to use?
2. Can you influence the amount of work assigned to you?
3. Can you influence decisions concerning the persons you will need to collaborate with?
4. Can decide when to be in contact with clients?
5. Can you influence decisions that are important for your work?

Pacing control in work

1. Can you set your own work pace?
2. Can you decide yourself when you are going to take a break?
3. Can you decide the length of your break?
4. Can you set your own working hours (flextime)?

Social climate in organization

What is the climate like in your work unit (colleagues and immediate superior)?

1. Encouraging and supportive?
2. Distrustful and suspicious?
3. Relaxed and comfortable?

## Supporting information

S1 Data File containing all data used. (SAV)

## Abbreviations

BMI: Body mass index
GAMM: Generalized additive mixed model
Max: Maximum
Min: Minimum
MSD: Musculoskeletal disorders
RHR: Relative heart rate
SD: Standard deviation
Sig: Significance
VAS: Visual analog scale
